# Supplementary material for: Enteral nutrition management in critically ill adult patients and its relationship with intensive care unit-acquired muscle weakness: A national cohort study
Source: PLoS One. 2023 Jun 7;18(6):e0286598. doi: 10.1371/journal.pone.0286598 (PMC10246809; doi:10.1371/journal.pone.0286598)
Supplement: S1 File — (PDF) [file pone.0286598.s001.pdf]

S1 File. Measurement tools: definitions and references

Body Mass Index: (BMI) is a method for estimating body fat mass. BMI is calculated by dividing the subject's mass by the square of his or her height (BMI= kilogram/ metres^2). Standard criteria are used to divide individuals into four groups: underweight (<18.5 kg/m2), normal (18.5-24.9 kg/m^2), overweight (25-29.9 kg/m2) or obese (≥30 kg/m^2).

Charlson comorbidity index is a marker for chronic disease burden that helps to predict the ten-year mortality for patients with a range of comorbid conditions. Charlson Comorbidity scores range from 0 to 33, with higher scores indicating a greater burden of chronic illness.

Charlson comorbidity index scoring:

1 point applied to each of the following: myocardial infarction, congestive heart failure, peripheral vascular disease, dementia, cerebrovascular disease, chronic lung disease, connective tissue disease, ulcer, chronic liver disease and diabetes; 2 points applied to each of the following: hemiplegia, moderate or severe kidney disease, diabetes with complication, tumor, leukemia, lymphoma; 3 points applied to each of the following: moderate or severe liver disease; 6 points applied to each of the following: malignant tumor, metastasis, AIDS.

Barthel Index for Activities of Daily Living is a measure of the independence of patient in their activities of daily living (with a higher score denoting a higher level of independence). The measurement was taken prior to admission, during an interview with the patient when possible or with the main carer.

Medical Research Council scale (MRC) is a validated scale for testing muscle strength**.** This scale requires patients´ cooperation and evaluates muscle strength on a scale of 0 (no muscle contraction) to 5 (full strength). To assess patients' level of cooperation and ability to participate

in testing, physiotherapists assessed eligible patients on a daily basis using Hermans' standardised commands as follows:

1. Open and close your eyes; 2. Look at me; 3. Open your mouth and put out your tongue; 4. Nod your head; 5. Raise your eyebrows after I have counted to five.

Physical examination of 3 muscle groups in each of the upper and lower limbs results in a composite or sum score of 60.

All physiotherapists participating in this study were trained in the use of this scale according to the protocol described by Hermans G., available at http://links.lww.com/CCM/A780 (accessed on 25th August 2018). Before the start of the training, a pilot test was conducted with simulated patients. This test obtained an inter-observer reliability of 0.697 [CI 95% 0.547 – 0.830] with only one reading of the protocol. During the present study, the MRC scale was measured every 7 days after the patient's awakening (RASS from 0 to -2). The assessment was conducted before the patient mobilized (for example, before hygiene-related activities). ICUAW was diagnosed for values lower than 48 out of 60 (the maximum score) in the first measure of MRC (baseline MRC).

ICU Mobility Scale (IMS) is a measure of mobility milestones for critically ill patients. It is a 10-point scale, ranging from 0 (patient immobile lying in bed) to 10 (independent ambulation). Some authors have categorized it as a binary system (<4 no mobilization and ≥4 mobilization) or as the following 4-level classification:

Category	Classification	Definition
0	Immobile	Lying in bed
1-2	Bed exercises or passive transfers. Low intensity	In bed mobilization (any activity undertaken whilst the patient is sitting or lying in bed such as rolling, bridging, upper-limb weight training, passive transfer to chair without weight bearing, such as in a sling lifter)

3-5	Actively moving in or out of bed. Moderate intensity	Active out of bed mobilization (sitting over the edge of the bed (dangling), standing, active transfer to chair with weight bearing, with or without assistance)
6-10	Marching on the spot or ambulating away from the bed-space. Higher intensity	Walking away from the bed space or marching on the spot with or without assistance

All professionals were trained to use the scales through an online platform with post-training evaluation.

References

World Health Organisation (WHO). Obesity and overweight. [Internet]. 2016 [cited 2020 Feb 18]; Available from: http://www.who.int/mediacentre/factsheets/fs311/en/.

Charlson ME y col. A new method of classifying prognostic comorbidity in longitudinal studies: development and validation. J Chronic Dis.1987; 40(5):373-83

Quan H, Li B, Couris CM, Fushimi K, Graham P, Hider P, et al. Updating and validating the Charlson comorbidity index and score for risk adjustment in hospital discharge abstracts using data from 6 countries. Am J Epidemiol 2011; 173(6): 676-82.

Shah S, Vanclay F, Cooper B. Improving the sensitivity of the Barthel Index for stroke rehabilitation. J Clin Epidemiol. 1989;42(8):703-709.

Hermans G. Assessment protocol of limb muscle strength in critically ill patients admitted to the ICU: the Medical Research Council Scale. Available in: http://links.lww.com/CCM/A780 (accessed april 2020).

Fan E, Ciesla ND, Truong AD, Bhoopathi V, Zeger SL, Needham DM. Inter-rater reliability of manual muscle strength testing in ICU survivors and simulated patients. Intensive Care Med 2010; 36(6): 1038-43.

De Jonghe B, Sharshar T, Lefaucheur JP, Authier FJ, Durand-Zaleski I, Boussarsar M, et al. Paresis acquired in the intensive care unit: a prospective multicenter study. JAMA 2002; 288: 2859-67.

Hodgson CL, Needham D, Haines K, Bailey M, Ward A, Harrold M, et al. Feasibility and inter-rater reliability of the ICU Mobility Scale. Heart Lung 2014; 43: 19–24.

Tipping CJ, Bailey MJ, Bellomo R, Berney S, Buhr H, Denehy L, et al. The ICU Mobility Scale Has Construct and Predictive Validity and Is Responsive. A Multicenter Observational Study. Ann Am Thorac Soc 2016; 13(6): 887-93.

Brock C, Marzano V, Green M, Wang J, Neeman T, Mitchell I, et al. Defining new barriers to mobilisation in a highly active intensive care unit -have we found the ceiling? An observational study. Heart Lung 2018; 47(4): 380-5.

Rebel A, Marzano V, Green M, Johnston K, Wang J, Neeman T, et al. Mobilisation is feasible in intensive care patients receiving vasoactive therapy: An observational study. Aust Crit Care 2019; 32(2): 139-46.

Hodgson CL, Bailey M, Bellomo R, Berney S, Buhr H, Denehy L, et al. Trial of Early Activity and Mobilization Study Investigators. A Binational Multicenter Pilot Feasibility Randomized Controlled Trial of Early Goal-Directed Mobilization in the ICU. Crit Care Med 2016; 44(6): 1145-52.
