# Supplementary material for: Enteral nutrition management in critically ill adult patients and its relationship with intensive care unit-acquired muscle weakness: A national cohort study
Source: PLoS One. 2023 Jun 7;18(6):e0286598. doi: 10.1371/journal.pone.0286598 (PMC10246809; doi:10.1371/journal.pone.0286598)
Supplement: S2 File — (PDF) [file pone.0286598.s002.pdf]

S1 File. Logistic Regression

First analysis: we investigated whether the onset of ICUAW can be explained by energy intake (kcal/kg/day) on days 3-7 of the ICU stay. We referred to this as the 'raw variable'.

Outcome variable: ICUAW YES/NO

Explanatory variable: energy

Second analysis: we again investigated whether the onset of ICUAW can be explained by energy intake (Kcal/Kg/day) on days 3-7 of the ICU stay, but corrected by a block of baseline variables (age, gender, BMI, Barthel and Charlson).

Outcome variable: ICUAW YES/NO

Explanatory variable: energy + baseline variables

Third analysis: we investigated whether the onset of ICUAW can be explained by energy intake (kcal/kg/day) on days 3-7of the ICU stay, but corrected by a block of variables recorded on ICU stay days 3-7 (days with CCRT, doses of ICUAW-related drugs and vasopressors, days with IMS ≥4, and days with moderate and severe hyperglycaemia).

Outcome variable: ICUAW YES/NO

Explanatory variable: energy + variables recorded on ICU stay days 3-7

Fourth analysis: we investigated whether the onset of ICUAW can be explained by energy intake (kcal/kg/day) on days 3-7 of the ICU stay , but corrected by all variables: baseline variables and variables recorded on ICU stay days 3-7.

Outcome variable: ICUAW YES/NO

Explanatory variable: energy + baseline variables+ variables recorded on ICU stay days 3-7

The same analysis was then performed with these variables: protein intake on days 3-7, days with overfeeding and days with protein >0.8 g/kg/day.

Logistic regression model for ICUAW and **mean energy on ICU days 3-7** (ESPEN guidelines)

	OR	95% confidence interval	p value
Mean Energy intake, raw	1.000	0.965-1.037	0.99
Mean Energy intake adjusting for baseline variables	1.007	0.969-1.046	0.72
Mean Energy intake adjusting for ICU stay variables	1.002	0.964-1.042	0.90
Mean Energy intake adjusting for all variables	1.008	0.967-1.050	0.71

Logistic regression model for ICUAW and **mean energy on ICU days 3-7** (ASPEN guidelines)

	OR	95% confidence interval	p value
Mean Energy intake, raw	1.000	0.963-1.039	1.00
Mean Energy intake adjusting for baseline variables	1.008	0.969-1.050	0.69
Mean Energy intake adjusting for ICU stay variables	1.003	0.964-1.044	0.88
Mean Energy intake adjusting for all variables	1.010	0.966-1.055	0.67

Logistic regression model for ICUAW and **mean protein intake on ICU days 3-7** (ESPEN guidelines)

	OR	95% confidence interval	p value
Mean protein intake, raw	1.146	0.504-2.607	0.75
Mean protein intake adjusting for baseline variables	1.207	0.513-2.838	0.67
Mean protein intake adjusting for ICU stay variables	1.196	0.500-2.865	0.69
Mean protein intake adjusting for all variables	1.212	0.482-3.049	0.68

Logistic regression model for ICUAW and **mean protein intake on ICU days 3-7** (ASPEN guidelines)

	OR	95% confidence interval	p value
Mean protein intake, raw	1.126	0.492-2.578	0.78
Mean protein intake adjusting for baseline variables	1.177	0.499-2.778	0.71
Mean protein intake adjusting for ICU stay variables	1.194	0.497-2.870	0.69
Mean protein intake adjusting for all variables	1.206	0.479-3.039	0.69

Logistic regression model for ICUAW and days with **overfeeding on ICU days 3-7** (ESPEN guidelines)

	OR	95% confidence interval	p value
Days with overfeeding, raw	1.085	0.934-1.261	0.29
Days with overfeeding adjusting for baseline variables	1.109	0.948-1.296	0.20
Days with overfeeding adjusting for ICU stay variables	1.106	0.945-1.294	0.21
Days with overfeeding adjusting for all variables	1.128	0.956-1.332	0.15

Logistic regression model for ICUAW and days with **overfeeding on ICU days 3-7** (ASPEN guidelines)

	OR	95% confidence interval	p value
Days with overfeeding, raw	0.997	0.835-1.191	0.97
Days with overfeeding adjusting for baseline variables	1.029	0.85-1.246	0.77
Days with overfeeding adjusting for ICU stay variables	0.997	0.831-1.196	0.98
Days with overfeeding adjusting for all variables	1.040	0.851-1.271	0.70

Logistic regression model for ICUAW and days with **protein >0.8 g/kg/day** (ICU days 3-7) (ESPEN guidelines)

	OR	95% confidence interval	p value
Days with protein >0.8 g/kg/day, raw	1.041	0.896-1.210	0.60
Days with protein >0.8 g/kg/day adjusting for baseline variables	1.054	0.903-1.231	0.51
Days with protein >0.8 g/kg/day adjusting for ICU stay variables	1.046	0.894-1.223	0.58
Days with protein >0.8 g/kg/day adjusting for all variables	1.048	0.890-1.234	0.572

Logistic regression model for ICUAW and days with **protein >0.8 g/kg/day** (ICU days 3-7) (ASPEN guidelines)

	OR	95% confidence interval	p value
Days with protein >0.8 g/kg/day, raw	1.008	0.869-1.169	0.92
Days with protein >0.8 g/kg/day adjusting for baseline variables	1.011	0.868-1.179	0.89
Days with protein >0.8 g/kg/day adjusting for ICU stay variables	1.012	0.867-1.181	0.88
Days with protein >0.8 g/kg/day adjusting for all variables	1.005	0.855-1.181	0.95
