# Supplementary figures and images for: Enteral nutrition management in critically ill adult patients and its relationship with intensive care unit-acquired muscle weakness: A national cohort study
Source: PLoS One. 2023 Jun 7;18(6):e0286598. doi: 10.1371/journal.pone.0286598 (PMC10246809; doi:10.1371/journal.pone.0286598)

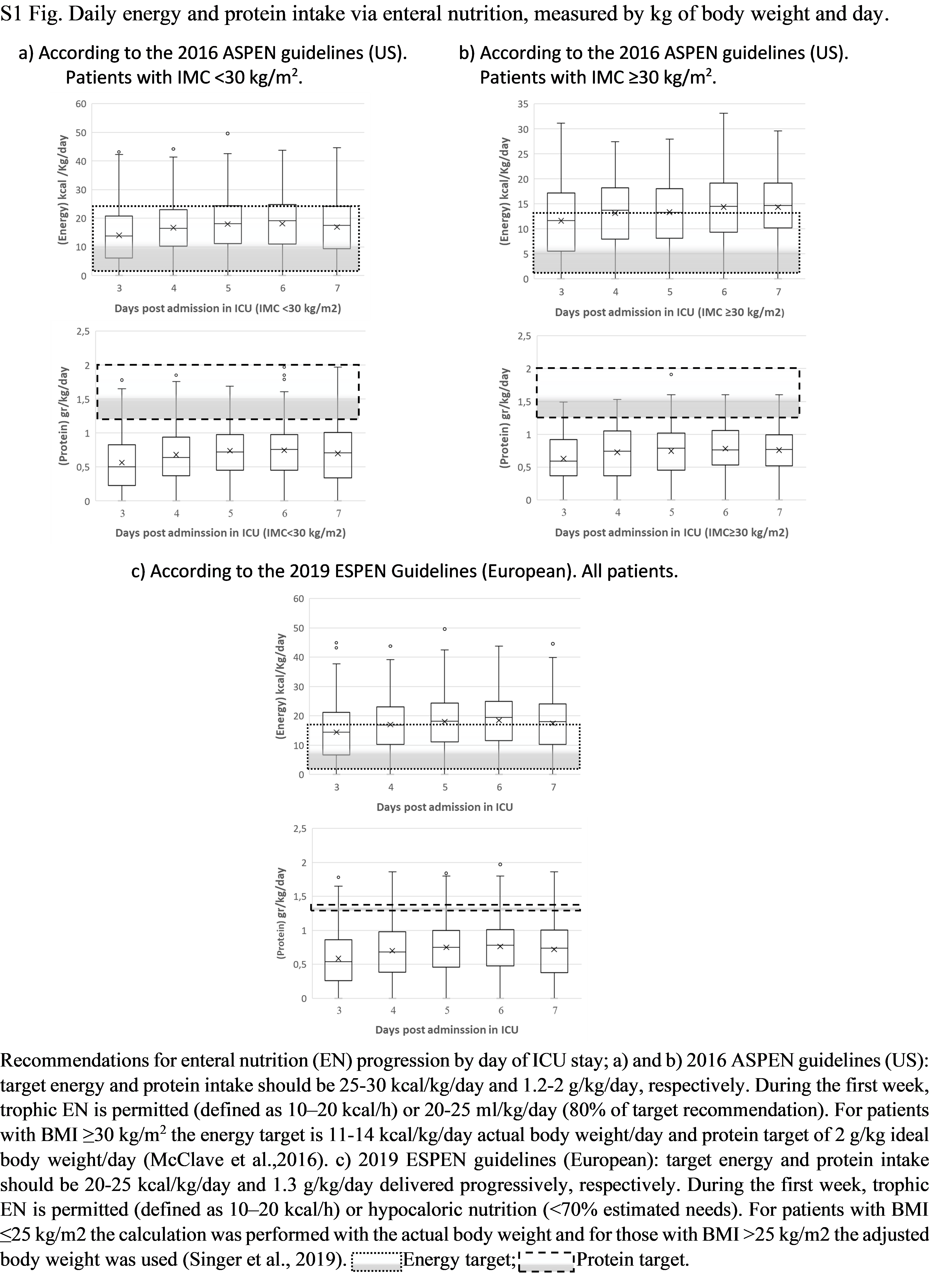

Supplement: S1 Fig — (TIF) [file pone.0286598.s003.tif]
