# Supplementary material for: Enteral nutrition management in critically ill adult patients and its relationship with intensive care unit-acquired muscle weakness: A national cohort study
Source: PLoS One. 2023 Jun 7;18(6):e0286598. doi: 10.1371/journal.pone.0286598 (PMC10246809; doi:10.1371/journal.pone.0286598)
Supplement: S1 Table — (PDF) [file pone.0286598.s004.pdf]

S1 Table. Energy and protein intake by target recommendation

ENERGY INTAKE days 3-7					
2016 ASPEN GUIDELINES (US) BMI <30 kg/m²		2016 ASPEN GUIDELINES (US) BMI ≥30 kg/m²		2019 ESPEN Guidelines (European)	
Variable	EN + Propofol % (pt/days)*	Variable	EN + Propofol % (pt/days)*	Variable	EN + Propofol % (pt/days)*
Below trophic feeding (< 10 kcal/h/day)	10.0% (122)	Below trophic feeding (< 10 kcal/h/day)	8% (30)	Below trophic feeding (< 10 kcal/h/day)	9.5% (151)
Trophic feeding (10-20 kcal/h/day)	7.0% (85)	Trophic feeding (10-20 kcal/h/day)	8.5% (32)	Trophic feeding (10-20 kcal/h/day)	7.3% (117)
Above trophic feeding but below target goal (>20 kcal/h/day <20 kcal/kg/day)	44.9% (548)	Above trophic feeding but below target goal (>20 kcal/h/day <11 kcal/kg/day)	19.5% (73)	Above trophic feeding but below target goal (>20 kcal/h/day <14 kcal/kg/day)	20.4% (325)
Target goal (20-25 kcal/kg/day)	18.4% (224)	Target goal (11-14 kcal/kg/day)	15.5% (58)	Target goal (14-17,5 kcal/kg/day)	12.6% (202)
Above target goal (>25 kcal/kg/day)	19.7% (241)	Above target goal (>14 kcal/kg/day)	48.5% (182)	Above target goal (>17,5 kcal/kg/day)	50.2% (800)
Overfeeding	12.5% (153)	Overfeeding	42.9% (161)	Overfeeding	43.1% (687)
**PROTEIN INTAKE days 3-7**					
2016 ASPEN GUIDELINES (US) BMI <30 kg/m²		2016 ASPEN GUIDELINES (US) BMI ≥30 kg/m²		2019 ESPEN Guidelines (European)	
Variable	EN % (pt/days)*	Variable	EN % (pt/days)*	Variable	EN % (pt/days)*
<0.5 g/kg/day	35.5% (433)	<0.5 g/kg/day	30.1% (113)	<0.5 g/kg/day	33.5% (535)
≥0.5 g/kg/day <0.8 g/kg/day	26.5% (323)	≥0.5 g/kg/day <0.8 g/kg/day	24.0% (90)	≥0.5 g/kg/day <0.8 g/kg/day	25.7% (410)
≥0.8 g/kg/day <1.2 g/kg/day	25.5% (312)	≥0.8 g/kg/day <1.2 g/kg/day	34.4% (129)	≥0.8 g/kg/day ≤1.3 g/kg/day	32.3% (514)
≥1.2 g/kg/day ≤2 g/kg/day	12.5% (152)	≥1.2 g/kg/day ≤2 g/kg/day	11.5% (43)	>1.3 g/kg/day ≤2 g/kg/day	8.5% (136)
> 2 g/kg/day	0.0% (0)	> 2 g/kg/day	0.0% (0)	> 2 g/kg/day	0.0% (0)

pt/days: patient/days; 2016 ASPEN guidelines (US): target energy and protein intake should be 25-30 kcal/kg/day and 1.2-2 g/kg/day, respectively. During the first week, trophic EN is permitted (defined as 10–20 kcal/h) or 20-25 ml/kg/day (80% of target recommendation). For patients with BMI ≥30 kg/m^2 the energy target is 11-14 kcal/kg/day actual body weight/day and the protein target is 2 g/kg ideal body weight/day (McClave et al., 2016); 2019 ESPEN guidelines (European): target energy and protein intake is 20-25 kcal/kg/day and 1.3 g/kg/day delivered progressively, respectively. During the first week, trophic EN is permitted (defined as 10–20 kcal/h) or hypocaloric nutrition (<70% estimated needs). Actual body weight is used for patients with BMI ≤25 kg/m^2 and adjusted body weight for BMI >25 kg/m^2. A protein intake of <0.5 g/kg/day is considered a low protein diet (Singer et al., 2019). Overfeeding is defined as “energy administration of 110% above the defined target” (Singer et al., 2019). *<0.001 according to Fisher test for comparison between the different categories.
