# Supplementary material for: Enteral nutrition management in critically ill adult patients and its relationship with intensive care unit-acquired muscle weakness: A national cohort study
Source: PLoS One. 2023 Jun 7;18(6):e0286598. doi: 10.1371/journal.pone.0286598 (PMC10246809; doi:10.1371/journal.pone.0286598)
Supplement: S2 Table — (PDF) [file pone.0286598.s005.pdf]

S2 Table. Other recommendations for enteral nutrition on ICU days 3 to 7

		Total<br>n (%)	ICUAW<br>n (%)	No-ICUAW<br>n (%)	p-value
Glycaemia<br><180mg/dl		1248 (78.9%)	605 (78.9%)	291 (85.8%)	0.006
Insulin received		336 (21.5%)	145 (19.3%)	78 (23.1%)	0.145
Cessations	None	439 (29.1%)	225 (31.3%)	94 (28.8%)	0.784*
	Once	427 (28.4%)	216 (30.1%)	103 (31.6%)	
	> once	640 (42.5%)	277 (38.6%)	129 (39.6%)	
GRV	<500 ml	1017 (95.3%)	477 (96.8%)	218 (94.0%)	0.108

%: percentage; n: number of patient-days; ICUAW: intensive care unit-acquired muscle weakness; * Calculations based on difference between 0-1 cessations vs more than 1 cessation; GRV: gastric residual volume.

Mean daily glycaemia was obtained and then categorized; Insulin given refers to rapid-acting insulin administered by intravenous infusion; Cessations include interruptions and pauses in enteral nutrition; Gastric residual volume was collected in the enteral tube drainage bag.
