# Supplementary material for: Enteral nutrition management in critically ill adult patients and its relationship with intensive care unit-acquired muscle weakness: A national cohort study
Source: PLoS One. 2023 Jun 7;18(6):e0286598. doi: 10.1371/journal.pone.0286598 (PMC10246809; doi:10.1371/journal.pone.0286598)
Supplement: Attachment — Submitted filename: R1_Response to Reviewers.docx [file pone.0286598.s006.docx]

Journal requirements:

When submitting your revision, we need you to address these additional requirements.

1. Please ensure that your manuscript meets PLOS ONE's style requirements, including those for file naming. The PLOS ONE style templates can be found at

<https://journals.plos.org/plosone/s/file?id=wjVg/PLOSOne_formatting_sample_main_body.pdf> and

<https://journals.plos.org/plosone/s/file?id=ba62/PLOSOne_formatting_sample_title_authors_affiliations.pdf>

Thank you very much for the indications. We have revised the document and adapted it to PlosOne standards.

2. Please provide additional details regarding participant consent. In the ethics statement in the Methods and online submission information, please ensure that you have specified what type you obtained (for instance, written or verbal, and if verbal, how it was documented and witnessed). If your study included minors, state whether you obtained consent from parents or guardians. If the need for consent was waived by the ethics committee, please include this information.

We have added details about participant consent to the manuscript in the Methods section (lines 157-158). In addition, we have made sure to include obtaining consent in the online submission information.

Our study did not include minors.

3. Thank you for stating the following financial disclosure:

“This work was supported by 2018 european federation of critical care nursing associations (EfCCNa) Research Awards.”

Please state what role the funders took in the study.  If the funders had no role, please state: "The funders had no role in study design, data collection and analysis, decision to publish, or preparation of the manuscript."

If this statement is not correct you must amend it as needed.

Please include this amended Role of Funder statement in your cover letter; we will change the online submission form on your behalf.

Thank you very much for your contribution and for the indication of the sentence to be added. We have modified the cover letter with this information.

4. Please include captions for your Supporting Information files at the end of your manuscript, and update any in-text citations to match accordingly. Please see our Supporting Information guidelines for more information: <http://journals.plos.org/plosone/s/supporting-information>.

Thank you very much for the indications. We have revised the document and adapted it to PlosOne standards.

[Note: HTML markup is below. Please do not edit.]

Reviewers' comments:

Reviewer's Responses to Questions

**Comments to the Author**

1. Is the manuscript technically sound, and do the data support the conclusions?

The manuscript must describe a technically sound piece of scientific research with data that supports the conclusions. Experiments must have been conducted rigorously, with appropriate controls, replication, and sample sizes. The conclusions must be drawn appropriately based on the data presented.

Reviewer #1: Yes

Reviewer #2: Partly

2. Has the statistical analysis been performed appropriately and rigorously?

Reviewer #1: Yes

Reviewer #2: N/A

3. Have the authors made all data underlying the findings in their manuscript fully available?

The [PLOS Data policy](http://www.plosone.org/static/policies.action#sharing) requires authors to make all data underlying the findings described in their manuscript fully available without restriction, with rare exception (please refer to the Data Availability Statement in the manuscript PDF file). The data should be provided as part of the manuscript or its supporting information, or deposited to a public repository. For example, in addition to summary statistics, the data points behind means, medians and variance measures should be available. If there are restrictions on publicly sharing data—e.g. participant privacy or use of data from a third party—those must be specified.

Reviewer #1: Yes

Reviewer #2: Yes

4. Is the manuscript presented in an intelligible fashion and written in standard English?

PLOS ONE does not copyedit accepted manuscripts, so the language in submitted articles must be clear, correct, and unambiguous. Any typographical or grammatical errors should be corrected at revision, so please note any specific errors here.

Reviewer #1: Yes

Reviewer #2: Yes

5. Review Comments to the Author

Please use the space provided to explain your answers to the questions above. You may also include additional comments for the author, including concerns about dual publication, research ethics, or publication ethics. (Please upload your review as an attachment if it exceeds 20,000 characters)

Reviewer #1: Reword the main objective so that it has a single action verb. For example, assess the incidence and determinants of ICUAW....

We agree with your comment. We have made the modification to the objective and it now has only one action verb.

Modifications have been made in the summary (line 47) and in the general text (line 127).

In abstract, methodology to be improved

We agree with your suggestion. We have completed the methodology section in the abstract (lines 50-52).

Methods
Specify year, country in methodology

We have added the year and country in methodology (line 142).

Specify demographic and clinical parameters, and how they were obtained

We have completed the variables section (in the methodology section), specifying the demographic and clinical parameters, as well as how they were obtained (lines 195-204). At the end of this section, it is detailed that the professionals who collected data were previously trained (lines 218-219).

Reviewer #2: Important clinical issue are discussed in the study under revision. I pointed out have some work aspects to improve the manuscript.

Major League:

In the introduction, the different ICUAW predictors are not sufficiently explained in order to reinforce the orientation of the problem. Explain the reasons for this choice of predictors based on evidence data.

We have reviewed the section of the introduction where factors related to ICUAW are considered. We have added a description of these factors in relation to the available evidence (lines 110-115).

In the discussion section, the incidence reported does not reflect the reality of the study (48% vs 68.9%), which constitutes a bias in the interpretation of the results and the different comparisons. The entire study population was not assessed on this criterion.

It is true that some of the patients collected could not obtain the ICUAW assessment because of the need for the patient to be awake in order to use the MRC scale (this limitation has been described in the limitations section).

Other authors who use the same tool have found the same problem. (Raurell-Torredá 2021; Eggmann 2020). In the same way, these results are in line with the systematic review by Appleton (Appleton 2015): “Approximately a quarter of patients were not able to comply with clinical evaluation and this may be responsible for potential underreporting of this condition. Patients unable to comply with clinical assessment tend to have a higher mortality rate and potentially have greater encephalopathy, both of which are associated with increased incidences of ICUAW”.

As pointed out in the Appleton’s systematic review (2015), the incidence of ICUAW is likely to be higher than that obtained by clinical assessment (such as MRC), is likely to be explained in part by higher rates of incomplete testing. The incidence of ICUAW is lower when it is diagnosed with a clinical assessment (413/1276, 32%, 95% CI 30–35%) than when it is diagnosed using electrophysiological techniques (749/1591, 47%, 95% CI 45–50%). As such, for the cases where the MRC assessment could not be conducted, it is likely that these patients would have been diagnosed with ICUAW if other techniques had been used, such as ultrasonography or electrophysiology. Furthermore, due to the lack of a formal training program or a standardized protocol to educate clinicians in this technique, an adequate training of the personnel in the 80 ICUs participants of the study was not feasible.

Additionally, it is worth mentioning that the MRC scale is the technique for the diagnosis of ICUAW recommended by the consensus of experts (Needham 2017).

The calculation of the incidence of ICUAW was obtained with those individuals for whom MRC could be measured.

Furthermore, it is important to note that if we use the whole population to calculate ICUAW, including those who could not be measured for MRC, we could have an erroneous result, as the missing patients have been considered as non-ICUAW (153/319=0.48 (48%)). This is contrary to Appelton's systematic review.

On the other hand, we have decided not to provide information from the general population so that readers would not find the tables confusing. However, if you find this information useful, we can add it so that you can see the results for the whole population. All tables are added below with an additional column providing the results for the whole population.

**References:**

Raurell-Torredà M, Arias-Rivera S, Martí JD, et al. Care and treatments related to intensive care unit-acquired muscle weakness: A cohort study. Aust Crit Care. 2021;34(5):435-445. doi:10.1016/j.aucc.2020.12.005

Eggmann S, Luder G, Verra ML, Irincheeva I, Bastiaenen CHG, Jakob SM. Functional ability and quality of life in critical illness survivors with intensive care unit acquired weakness: A secondary analysis of a randomised controlled trial. PLoS One. 2020;15(3):e0229725. Published 2020 Mar 4. doi:10.1371/journal.pone.0229725

Appleton RT, Kinsella J, Quasim T. The incidence of intensive care unit-acquired weakness syndromes: A systematic review. J Intensive Care Soc. 2015 May;16(2):126-136

Needham DM, Sepulveda KA, Dinglas VD, Chessare CM, Friedman LA, Bingham CO 3rd, et al. Core outcome measures for clinical research in acute respiratory failure survivors: an international modified Delphi consensus study. Am J Respir Crit Care Med. 2017;196(9):1122-1130. doi: 10.1164/rccm.201702-0372OC.

Table 1. General characteristics of the study population

|  | | Patient  (receiving EN alone at least on days 3-7) n=319 (100%) | Patients with EN who developed ICUAW | Patients with EN who did NOT develop ICUAW | p value |
| --- | --- | --- | --- | --- | --- |
| **Total n (%)** | | 319 (100%) | 153 (48.0%) | 69 (21.6%) |  |
| Gender  n (%) | Female | 90 (28.2%) | 52 (34.0%) | 14 (20.3%) | 0.041 |
| Age, years  Median [IQR] | | 66 [54-76] | 68.0 [55.0-76.0] | 63.0 [47.5-74.5] | 0.079 |
| Dx. on Admission  n (%) | Sepsis | 58 (18.2%) | 32 (20.9%) | 9 (13.0%) | <0.001 |
|  | Trauma | 28 (8.8%) | 10 (6.5%) | 7 (10.1%) | 0.467 |
|  | Neurosurgery | 13 (4.1%) | 4 (2.6%) | 0 (0%) | - |
|  | Cardiovascular surgery | 24 (7.5%) | 15 (9.8%) | 4 (5.8%) | 0.012 |
|  | Other surgeries | 32 (10.0%) | 18 (11.8%) | 8 (11.6%) | 0.050 |
|  | Overdose | 8 (2.5%) | 3 (2.0%) | 2 (2.9%) | 0.655 |
|  | Other medical patients | 156 (48.9%) | 71 (46.4%) | 39 (56.5%) | 0.002 |
| BMI (kg/m^2^)  Median [IQR] | | 27.7 [24.2-29.4] | 27.1 [24.3-30.3] | 26.7 [24.0-30.8] | 0.752 |
| BMI^a^  n (%) | Underweight | 2 (0.6%) | 2 (1.3%) | 0 (0.0%) | - |
|  | Normal | 101 (31.7%) | 46 (30.1%) | 23 (33.3%) | 0.006 |
|  | Overweight | 141 (44.2%) | 66 (43.1%) | 24 (34.8%) | <0.001 |
|  | Obese | 75 (23.5%) | 39 (25.5%) | 22 (31.9%) | 0.030 |
| Barthel  Median [IQR] | | 100 [95-100] | 100 [95-100] | 100 [95-100] | 0.933 |
| Charlson index  Median [IQR] | | 5.0 [2.0-7.0] | 5.0 [2.0-7.0] | 4.0 [1.0-6.0] | 0.247 |
| APACHE II^d^  Median [IQR] | | 22 [16-27] | 23 [18-28] | 21 [16-27] | 0.537 |
| ICU stay in days  Median [IQR] | | 18 [13-29] | 22.0 [15.0-34.5] | 15.0 [11.0-22.0] | <0.001 |

Table 2. ICU variables, by ICUAW onset on days 3-7 of ICU stay

|  | Patient  (receiving EN alone at least on days 3-7) n=319 (100%) | Patients with EN who developed ICUAW  n=153 (48.0%) | Patients with EN who did NOT develop ICUAW  n=69 (21.6%) | p value |
| --- | --- | --- | --- | --- |
| IMS≥4 | 0.0 [0.0-0.0] / 0.04±0.23 | 0.0 [0.0-0.0] / 0.02±0.14 | 0.0 [0.0-0.0] / 0.12±0.44 | <0.001 |
| Days with CRRT | 0.0 [0.0-0.0]/ [0-4] | 0.0 [0.0-0.0]/ [0-2] | 0.0 [0.0-0.0]/ [0-4] | 0.327 |
| Days according to Airway management  - IMV  - no IMV | 5.0[5.0-5.0]  0.0[0.0-0.0] | 5.0[5.0-5.0]  0.0[0.0-0.0] | 5.0[4.0-5.0]  0.0[0.0-1.0] | 0.032  0.028 |
| ICUAW-related drugs (mg) | 87.0 [0.0-335.0] | 60.0 [0.0-307.4] | 0.0 [0.0-240.0] | 0.111 |
| Vasopressors (mg) | 21.9 [0.0-129.3] | 24.2 [0.0-123.5] | 7.7 [0.0-39.2] | 0.029 |
| Moderate hyperglycaemia (rate) | 9.1 [0.0-21.1] | 5.6 [0.0-20.9] | 5.9 [0.0-17.7] | 0.386 |
| Severe hyperglycaemia (rate) | 0.0 [0.0-15.6] | 0.0 [0.0-18.0] | 0.0 [0.0-11.5] | 0.223 |

Table 3. Energy and protein intake via EN and association with ICUAW on ICU days 3-7

|  | Patients receiving EN on days 3-7  n=319 (100%) | Patients with EN who developed ICUAW  n=153 (48.0%) | Patients with EN who did NOT develop ICUAW  n=69 (21.6%) | p value |
| --- | --- | --- | --- | --- |
| **Energy. 2016 ASPEN guidelines** |  |  |  |  |
| Energy (BMI <30 kg/m^2^) | 16.8 [11.3-22.2] | 16.8 [10.6-22.4] | 16.2 [11.1-22.5] | 0.899 |
| Overfeeding (BMI <30 kg/m^2^)  n (%) | 22 (9%) | 10 (8.8%) | 5 (10.6%) | 0.555 |
| Days with overfeeding on (BMI <30 kg/m^2^) | 0.0 [0.0-0.0] | 0.0 [0.0-0.0] | 0.0 [0.0-0.0] | 0.998 |
| Energy (BMI >30 kg/m^2^) | 13.0 [9.3-17.6] | 12.6 [9.1-18.3] | 12.0 [9.7-17.2] | 0.988 |
| Overfeeding (BMI >30 kg/m^2^)  n (%) | 29 (38.7%) | 12 (30.8%) | 8 (36.4%) | 0.778 |
| Days with overfeeding on days 3-7 (BMI >30 kg/m^2^)  Median [IQR] | 2.0 [0.0-4.0] | 2.0 [0.0-4.0] | 1.5 [0.0-3.0] | 0.787 |
| **Energy. 2019 ESPEN guidelines** |  |  |  |  |
| Energy | 17.5 [11.8-22.6] | 16.7 [10.7-23.0] | 15.9 [12.0-22.4] | 0.916 |
| Overfeeding  n (%) | 131 (41.1%) | 62 (40.5%) | 26 (37.7%) | 0.767 |
| Days with overfeeding | 2 [0-4] | 2 [0-4] | 1 [0-3] | 0.330 |
| **Protein.** **2016 ASPEN guidelines** |  |  |  |  |
| Prot (BMI <30 kg/m^2^) | 0.66 [0.46-0.88] | 0.65 [0.46-0.89] | 0.69 [0.44-0.91] | 0.873 |
| Days with protein >0.8 gr/kg/day (BMI <30 kg/m^2^) | 1.5 [0-4] | 1.0 [0.0-3.3] | 2.0 [0.0-4.0] | 0.598 |
| Protein (BMI >30 kg/m^2^) | 0.77 [0.50-0.94] | 0.77 [0.48-0.99] | 0.69 [0.52-0.87] | 0.409 |
| Days with protein >0.8 gr/kg/day (BMI >30 kg/m^2^) | 3.0 [0.0-4.0] | 3.0 [0.0-4.0] | 1.0 [0.0-3.0] | 0.238 |
| **Protein. 2019 ESPEN guidelines** |  |  |  |  |
| Protein | 0.69 [0.47-0.90] | 0.69 [0.46-0.92] | 0.68 [0.46-0.91] | 0.793 |
| Days with protein >0.8 gr/kg/day | 2 [0-4] | 2 [0-4] | 2 [0-4] | 0.654 |

Minor comments:
line 104: EN is already defined in line 97.

Thank you for your correction. We have made the change.

Lines 173-220: Write this section in simple, understandable text instead of listing items.

We have revised this section. We believe that it is now in a simpler and more understandable form to read.

Discussion: Most of the information can be given by just adopting the numbered reference since the names of the authors appear repeatedly. Review the writing style at this level.

We have revised the wording of the discussion section. We have removed the names of the authors, leaving only the bibliographical reference.

6. PLOS authors have the option to publish the peer review history of their article ([what does this mean?](https://journals.plos.org/plosone/s/editorial-and-peer-review-process#loc-peer-review-history)). If published, this will include your full peer review and any attached files.


If you choose “no”, your identity will remain anonymous but your review may still be made public.


**Do you want your identity to be public for this peer review?** For information about this choice, including consent withdrawal, please see our [Privacy Policy](https://www.plos.org/privacy-policy).

Reviewer #1: **Yes:** Guy Roussel Takuissu Nguemto

Reviewer #2: No
